# Cheyyur Ramaswamy Sundararajan

**DOI:** 10.4103/0970-0358.73423

**Published:** 2010

**Authors:** K. S. Shekar, B. A. Anantharam

**Affiliations:** Chairman, The Bangalore Hospital, Bangalore; 1 Director Medical Services - Annasawmy Mudaliar General Hospital, Bangalore, Honorary Consultant Plastic Surgeon & Head of Department, Plastic Reconstructive and Hand Surgery, St Martha’s Hospital, Bangalore & Sri Sathya Sai General Hospital, Whitefield, Bangalore

Professor Cheyyur Ramaswamy Sundararajan, popularly known to his students, colleagues, his friends in and outside the speciality of plastic surgery, and many of his fraternal associates as CRS, an acronym, which he quietly accepted (considering his disapproval of medical acronyms), was the “ultimate” guru.

A scion of the Cheyyur Ramaswamy family and considered the “baby” of the family (the only way he was addressed by his mother till her death) was induced by traditional family values of paternal guidance and advice. He was very keen on joining the army, having been an under officer during his intermediate days, but his father insisted on him taking up medicine. So much the better for medicine and for plastic surgery too!

But after his intermediate, he went on to complete his B.Sc. at Presidency College and then returned to renew his application for entry into the medical college which he did with renewed vigour. An alumnus of Madras Medical College, he realised his leaning towards surgery even during his student days “I always felt I wanted to become as surgeon”. Completing his Masters in General Surgery at Madras Medical College where he trained under Dr. U. Mohan Rao, he went on to serve the then Presidency of Madras and later the state of Madras, doing time and assistantship under late Dr. U. Mohan Rao, late Prof. Atmaram Rao and late Prof. H. Harirajan.

He was hand picked by the late Dr. V. R. Thayumanswami, the then Director of Medical Education, to be trained in the speciality of plastic surgery, and that too, in the only centre which was then available in India for formal training in plastic surgery at Nagpur under the legendary Prof. C. Balakrishnan.

Indeed it must have been a privilege to have been selected by the legendary Dr. V. R. Thayumanswami, a great soul who had that uncanny sense to pick and bring out the best of talents available for the development of specialties and units in the state of Madras, for it was this vision and wisdom that saw the setting up of the three premier units of plastic surgery in the state.

On the completion of his training under Prof. C. Balakrishnan, under the “Gillies tradition”, Prof. CRS returned to Madras with his MCh as the first indigenous surgeon to be awarded this post doctoral qualification, and that too from any specialty, in the country. Recognition for this training came only later. He was designated to head the first formal Department of Plastic Surgery in South India with training status. The training he imparted was as meticulous as the training he received—planning sessions, lectures, teaching sessions (some extending in the early days into the late evenings), journal readings and trainee participation to acquire teaching skills.

Marriage, in the meantime, to Rukmini (Amma to us), from the M. Bhaktavatsalam family, was to add a new meaning. (His father was leader of the opposition and father-in-law the leader of the house; they were always good friends in all matters but politics!)

Having set up the first formal department at Madras Medical College, initially as reader and then occupying the professorial chair as the head of the department, he chose, selected and trained 30 plus surgeons as plastic surgeons. He continued indeed to be very proud of every one of his trainees, who were able to qualify, with extreme satisfaction that many of them have done indeed very well for themselves and for the speciality.

It was generally believed that Dr. C. R. Sundararajan never used his enormous clout for personal enrichment. He remained just a reader in plastic surgery for many years and waited for his turn for promotion. From today’s standard, with the “clout” he had with the government, he never asked for any favours, either for himself or for anyone else. Often people wondered why he should be so strict to dislike being labelled as someone’s son or son-in-law.

His integrity was recognised by the Madras State Government because it finally requested him to Chair the selection committee for admittance to the Undergradate courses (MBBS) in the state. During the years when he was the Chair, Selection Committee, there were no writ petitions or if there were any, they were never upheld. Even till his end, if there was any problem in any of the professional organisations that he was connected with, he was consulted for his considered advice and guidance to solve the problems.

Having sat as an examiner at many of the universities, at times alongside his own preceptor, he earned a reputation as one of the fairest of examiners. Respected for his sagacity and brief communications which were usually typed by himself on his portable Remington or otherwise hand written (in a script characteristic to him), The Association of Plastic Surgeons of India (APSI) honoured him by electing him the President for 1975.

He later steered the course of the Association of Surgeons of India (ASI) as its President in 1985. His contributions to the field of Medicine in general and to the development of Plastic Surgery in particular brought him further recognition in 1989 when he was awarded the prestigious “Dr. BC Roy Award”.

Having retired with great satisfaction, he continued to be active academically and professionally by attending many a teaching sessions whenever possible.

Post retirement, he accepted an assignment as Medical Superintendent of the Medical College and Hospital at Chidambaram.

For us, he was the ultimate Guru. He moulded our character while he taught us the principles of plastic surgery. He exemplified by personal conduct. He never mixed private practice with hospital work. Honest to the core and punctual, he never discussed local or state or professional politics. He treated every one of his colleagues and trainees with respect and equanimity.

Deeply religious, he began each day with a focussed recitation of scriptures and holy writings. His preoccupation in the last few years was to renovate and rejuvenate the temple of his village, Cheyyur, with a splendid gopuram and general upliftment as a personal commitment.

The joy of reminiscing on this great friend, philosopher, guide and brother (yes a Brother Freemason too) is only to share the uniqueness of the association which has been totally impressionable and everlasting.

He was the man who talked the walk—one who preached what he practiced. This tribute can truly never do complete justice to the man that he was.

Prof. C. R. Sundararajan passed away in 2008 making many of us, in a sense, orphans. We live on his reflected glory.

**Figure 1:**
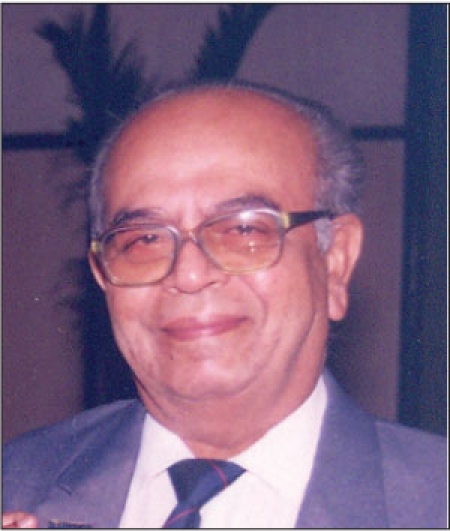
Prof. Cheyyur Ramaswamy Sundararajan

